# Latent profiles of HPV vaccine-related health beliefs and their associations with vaccination intention among college students: a global health perspective

**DOI:** 10.3389/fpubh.2026.1918113

**Published:** 2026-07-28

**Authors:** Ya Liu, Haiming Cao, Jiaqi Li, Weiwei Sun, Yunyu Zhang, Cuicui Meng, Nannan Zhang, Xia Li, Jingli Wu, Yiyi Zhuang, Wenming Cao

**Affiliations:** 1 Department of Gynecology, Shandong First Medical University Affiliated Heze Hospital (Heze Municipal Hospital), Heze, Shandong, China; 2 Department of Hepatobiliary Surgery, Zhuhai People's Hospital (The Affiliated Hospital of Beijing Institute of Technology, Zhuhai Clinical Medical College of Jinan University), Zhuhai, Guangdong, China; 3 Department of Gynecology, Pingshan District Central Hospital of Shenzhen, Shenzhen, Guangdong, China; 4 Health Industry Department, College of Health Industry, Xiamen Donghai Vocational and Technical College, Xiamen, Fujian, China

**Keywords:** college students, e-health literacy, health belief model, HPV vaccine, human papillomavirus, latent profile analysis, vaccination intention

## Abstract

**Purpose:**

Human papillomavirus (HPV) vaccination is an effective strategy for preventing cervical cancer and other HPV-related diseases, yet uptake among young adults remains suboptimal. College students may hold heterogeneous vaccine-related beliefs that are not fully captured by variable-centered analyses. This study aimed to identify latent profiles of HPV vaccine-related health beliefs among Chinese college students and examine their associations with HPV vaccine knowledge, social support and perceived service accessibility, e-health literacy, vaccination intention, and uptake.

**Methods:**

A cross-sectional survey was conducted among 2,755 Chinese college students recruited from five universities across Shandong and Guangdong provinces. HPV vaccine-related health beliefs were measured across five Health Belief Model dimensions: perceived susceptibility, perceived severity, perceived barriers, perceived benefits, and self-efficacy. The Information-Motivation-Behavioral Skills model guided the selection of HPV vaccine knowledge, social support and perceived service accessibility, and e-health literacy as correlates. Latent profile analysis was used to identify belief-based subgroups. Between-profile differences were examined using *t*-tests and chi-square tests, with effect sizes reported. Logistic regression was used to identify factors associated with membership in the more favorable profile.

**Results:**

Two profiles were identified: a high-barrier/moderate-benefit profile (74.0%) and a high-benefit/low-barrier profile (26.0%). Compared with the high-barrier/moderate-benefit profile, the high-benefit/low-barrier profile had higher HPV vaccine knowledge (6.54 vs. 4.61), social support and perceived service accessibility (3.55 vs. 3.26), e-health literacy (4.00 vs. 3.51), vaccination intention (3.96 vs. 3.43), and vaccination uptake (24.6% vs. 15.3%; all *p* < 0.001). Female sex, active information seeking, higher HPV vaccine knowledge, and higher e-health literacy were associated with greater odds of belonging to the high-benefit/low-barrier profile. E-health literacy showed the strongest adjusted association.

**Conclusion:**

Chinese college students showed distinct HPV vaccine-related health belief profiles. Most students recognized some benefits of HPV vaccination but reported substantial barriers. HPV vaccine knowledge, active information seeking, and e-health literacy were associated with more favorable belief profiles and may be considered potential targets for future health education interventions. Given the cross-sectional design, causal relationships cannot be inferred.

## 1 Introduction

Human papillomavirus (HPV) infection is one of the most common sexually transmitted infections worldwide. Persistent infection with high-risk HPV types is the necessary cause of most cervical cancer and is also associated with other anogenital and oropharyngeal diseases (1). HPV vaccination represents a significant primary prevention strategy with the capacity to substantially reduce the burden of HPV-related diseases (2). HPV vaccination is a core component of the World Health Organization’s global strategy to accelerate the elimination of cervical cancer as a public health problem (3, 4). The enhancement of HPV vaccination rates among young people represents a pivotal public health objective on a global scale, including in China, where the prevalence of cervical cancer remains considerable.

Despite the availability of safe and effective HPV vaccines, global vaccination coverage remains suboptimal, particularly in low- and middle-income countries (LMICs) where the cervical cancer burden is disproportionately high. While a number of high-income countries have attained considerable vaccination coverage through the implementation of school-based programs and national immunization policies, numerous LMICs persistently encounter significant challenges, including constrained financial resources, inadequate healthcare infrastructure, and competing health priorities (5, 6). China, as the world’s largest middle-income country, represents a particularly instructive case in this regard. The introduction of HPV vaccines is a relatively recent development, and the financial burden of vaccination is predominantly shouldered by the individual, rather than being incorporated into the national immunization program. Significant regional disparities in vaccine accessibility have been identified (7). It is imperative to comprehend the belief systems underpinning vaccine hesitancy among young adults in this context, as this can offer valuable insights for other countries facing similar challenges in expanding HPV vaccination coverage (8, 9).

It is widely acknowledged that college students are at a critical stage in their lives with regard to sexual and reproductive health education, independent health information seeking and vaccination decision-making (10). Although awareness of HPV vaccination has increased in many settings, knowledge, beliefs, intention and actual uptake may remain inconsistent. Some students may recognize that HPV vaccination is beneficial but still hesitate because of concerns about cost, safety, adverse reactions, effectiveness, access to vaccination services or social misunderstanding (11, 12). Others may have fewer concerns and stronger confidence in vaccination. Treating college students as a homogeneous group may therefore obscure important differences in vaccine-related beliefs and intervention needs. From a public health education perspective, understanding this heterogeneity is essential for designing differentiated health promotion interventions that move beyond one-size-fits-all awareness campaigns.

Previous studies among Chinese college students have examined HPV vaccine awareness, knowledge, willingness, perceived barriers, and uptake, providing important evidence on factors associated with vaccination intention and behavior. These studies have shown that HPV vaccination intention and uptake are associated with factors such as sex, medical background, vaccine knowledge, perceived safety, cost concerns, and access to vaccination services (13). However, most existing studies have focused on individual predictors rather than on the combined patterns of health beliefs within students. As a result, less is known about whether distinct subgroups of students hold different configurations of perceived benefits, barriers, susceptibility, severity, and self-efficacy. The present study contributes to this literature by using latent profile analysis to identify such subgroups and by examining how they differ in knowledge, social support, e-health literacy, intention, and uptake.

The Health Belief Model (HBM) provides a useful framework for understanding preventive health behavior (14, 15). According to this model, health behavior may be shaped by perceived susceptibility, perceived severity, perceived barriers, perceived benefits and self-efficacy. In the context of HPV vaccination, students may be more willing to receive the vaccine if they perceive HPV infection and HPV-related diseases as relevant and serious, recognize the benefits of vaccination, perceive fewer barriers and have greater confidence in the effectiveness and feasibility of vaccination (16–18). The HBM is particularly valuable for health education and promotion because its constructs are modifiable through targeted educational interventions. For example, addressing perceived barriers through clear communication about vaccine safety and cost, or enhancing perceived benefits through evidence-based messaging. The HBM is complemented by the Information-Motivation-Behavioral Skills (IMB) model, which offers a supplementary framework for understanding how knowledge, social influences, and self-efficacy interact to shape health behaviors. The IMB model posits that health behavior is a function of health-related information, personal and social motivation, and behavioral skills. This framework is particularly relevant for understanding the roles of HPV knowledge, social support, and e-health literacy (EHL) in vaccine decision-making (19, 20).

The HBM and IMB models were integrated because they address complementary aspects of HPV vaccination decision-making. The HBM captures students’ subjective beliefs about risk, severity, benefits, barriers, and self-efficacy, which were used as indicators for latent profile analysis. The IMB model emphasizes information, motivation, and behavioral skills. In the present study, HPV vaccine knowledge represented information, social support reflected social motivation, and e-health literacy reflected the ability to obtain, evaluate, and use health information in digital environments. HBM was used to define belief profiles, whereas IMB helped guide the selection of correlates that may distinguish between profiles. The integrated theoretical framework combining the HBM and IMB models is illustrated in Supplementary Figure 1.

Latent profile analysis (LPA) is a person-centered statistical method used to identify unobserved subgroups based on patterns of continuous indicators (21, 22). In comparison with conventional variable-centered analyses, LPA has the capacity to discern whether students with disparate combinations of HPV vaccine-related health beliefs are present within the same population (23, 24). The identification of such profiles has the potential to inform the development of tailored health education strategies and more precise vaccination promotion.

Despite the extensive research conducted on HPV vaccination in Western populations, the applicability of these findings to Asian populations, particularly Chinese college students, remains uncertain. This is due to cultural differences in health beliefs, varying healthcare systems, and distinct vaccine accessibility patterns (25, 26). The unique context of China, where HPV vaccine uptake remains suboptimal despite a high cervical cancer burden, highlights the need for a deeper understanding of the belief structures that underpin vaccine hesitancy among young adults. This understanding is crucial for the development of contextually relevant interventions (27, 28).

In light of the above, this study had three specific objectives: to identify distinct latent profiles of HPV vaccine-related health beliefs among Chinese college students using a person-centered approach; to compare these profiles in terms of HPV vaccine knowledge, social support, EHL, vaccination intention (VI), and actual vaccination uptake; and to explore factors associated with membership in the more positive health belief profile, with particular attention to the role of EHL as a potentially modifiable target for intervention.

## 2 Materials and methods

### 2.1 Study design and participants

This cross-sectional questionnaire-based study was conducted between September and December 2024. Participants were recruited from five universities across Shandong and Guangdong provinces using a multi-stage convenience sampling strategy. The participating universities included different institutional types, including comprehensive, medical, and technical universities. Within each university, students from different grade levels and disciplines were invited through class-based and university-based online survey channels.

Inclusion criteria were: currently enrolled full-time college student; able to understand and complete the questionnaire independently; and willing to provide informed consent.

Exclusion criteria were: incomplete questionnaire responses; logically inconsistent responses; and duplicate submissions.

A total of 3,022 questionnaires were collected, of which 2,755 were valid, yielding a valid questionnaire rate of 91.2%. The questionnaire collected information on sociodemographic characteristics, HPV vaccine awareness and uptake, HPV vaccine-related knowledge, health beliefs, social support, EHL and HPV VI. The sample size exceeded the recommended minimum of 500 for LPA and provided sufficient statistical power to detect medium effect sizes in logistic regression (based on the rule of 10 events per predictor variable). The participant recruitment and analysis process is summarized in Supplementary Figure 2.

### 2.2 Survey instruments

The questionnaire was developed based on the HBM and IMB models and adapted from established instruments and previous HPV vaccination studies (14). Health belief items were informed by HBM-based vaccination research, e-health literacy items were adapted from the eHealth Literacy Scale (29), and HPV vaccine knowledge items were adapted from previous studies conducted among Chinese populations. The questionnaire was reviewed for clarity, cultural appropriateness, and relevance to the HPV vaccination context among Chinese college students. Internal consistency was evaluated using Cronbach’s alpha for Likert-type scales and KR-20 for dichotomously scored knowledge items. The full questionnaire is provided in Supplementary Table 1, and variable coding, scoring rules, score ranges, and interpretation of each construct are shown in Supplementary Table 2.

#### 2.2.1 Sociodemographic and HPV-related characteristics

Sociodemographic variables included sex, discipline, grade, age group, residence, monthly living expenses, family economic status and age at first sexual intercourse. HPV-related variables included whether participants had heard of HPV vaccine, whether they had received at least one dose of HPV vaccine and whether they had actively searched for HPV vaccine-related information.

#### 2.2.2 HPV vaccine knowledge

HPV vaccine knowledge was measured using nine knowledge items. Each item was scored as 1 for a correct answer and 0 for an incorrect or uncertain answer. Total scores ranged from 0 to 9, with higher scores indicating greater HPV vaccine knowledge. The nine items covered recommended vaccination age, vaccination after 45 years of age, prevention of cervical cancer, vaccination for both men and women, the need for screening after vaccination, the coverage of the nine-valent vaccine, vaccination before first sexual intercourse, vaccination after sexual intercourse, and whether HPV vaccine can treat HPV infection. Because the nine HPV vaccine knowledge items were dichotomously scored as correct or incorrect/uncertain, internal consistency was assessed using the Kuder–Richardson Formula 20 (KR-20), which is appropriate for binary-coded items. The KR-20 coefficient was 0.78, exceeding the commonly accepted threshold of 0.70 for adequate internal consistency, indicating acceptable reliability for group-level comparisons.

#### 2.2.3 HPV vaccine-related health beliefs

The beliefs about the HPV vaccine were assessed using the HBM and divided into five parts: perceived susceptibility, perceived severity, perceived barriers, perceived benefits and self-efficacy. Perceived susceptibility included three items assessing perceived risk of HPV infection, cervical cancer, and sexually transmitted diseases. Perceived severity included three items reflecting perceived health, marital, psychological, and economic consequences of HPV infection or cervical cancer. While the original HBM focuses primarily on health and economic consequences, we expanded the assessment to include marital and psychological consequences to better capture the specific concerns prevalent among Chinese college students regarding HPV infection, reflecting cultural considerations around reproductive health and social stigma. Perceived barriers included three items reflecting concerns about vaccine cost, safety, effectiveness, and adverse reactions. Perceived benefits included three items reflecting perceived benefits of HPV vaccination in reducing HPV infection, cancer, and genital warts. Self-efficacy included two items reflecting beliefs about the durability and relative effectiveness of HPV vaccination. All health belief items were scored on a 5-point Likert scale from 1 to 5. Mean scores were calculated for each dimension, with higher scores indicating higher levels of the corresponding belief dimension.

#### 2.2.4 Social support

Social support and perceived service accessibility were measured using four items assessing encouragement and accompaniment from family members, recommendations from classmates or friends, advice from school doctors or other medical personnel and perceived ease of appointment for HPV vaccination at community health service centers. Items were scored from 1 to 5, and the mean score was used. Higher scores indicated greater perceived social support and service accessibility. Confirmatory factor analysis supported the single-factor structure of the social support and service accessibility measure (CFI = 0.97, RMSEA = 0.05), supporting its use as a composite indicator of the supportive environment for vaccination.

#### 2.2.5 E-health literacy

EHL was measured using eight items assessing students’ ability to search for, understand, obtain, evaluate and use online health information. Items were scored from 1 to 5, and the mean score was used. Higher scores indicated greater EHL.

#### 2.2.6 HPV vaccination intention and uptake

HPV VI was measured using four items assessing willingness to consult about HPV vaccination through social media, planning to receive HPV vaccination, confidence in receiving HPV vaccination and willingness to recommend HPV vaccination to friends or relatives. Items were scored from 1 to 5, and the mean score was used. Higher scores indicated stronger vaccination intention. HPV vaccination uptake was measured by whether participants had received at least one dose of HPV vaccine.

### 2.3 Statistical analysis

All statistical analyses were performed using SPSS 26.0 and Mplus 8.3 for confirmatory factor analyses. Categorical variables were summarized as frequencies and percentages, and continuous variables were summarized as means and standard deviations (SDs). Internal consistency was evaluated using Cronbach’s alpha. Questionnaires with incomplete responses, logically inconsistent answers, or duplicate submissions were excluded before analysis. Because only complete valid questionnaires were retained, no imputation was performed. For continuous variables, distributions were inspected before group comparisons. Independent-samples *t*-tests were used for continuous variables, and chi-square tests were used for categorical variables. Cohen’s *d* was reported for *t*-tests, and Cramér’s *V* was reported for chi-square tests to evaluate effect size.

LPA was performed using five health belief dimensions: perceived susceptibility, perceived severity, perceived barriers, perceived benefits and self-efficacy. Models with two to six profiles were estimated. The optimal number of profiles was determined based on the Bayesian Information Criterion (BIC), log-likelihood, entropy, class size, Vuong–Lo–Mendell–Rubin likelihood ratio test (VLMR), and theoretical interpretability. For the VLMR test, a non-significant result for the k-profile model indicates that adding one additional profile does not significantly improve model fit compared with the k-1 profile model. After the optimal number of profiles was determined, profiles were labelled according to their mean patterns across the five health belief dimensions.

To address potential multicollinearity between “heard of HPV vaccine” and “HPV vaccine knowledge” in the logistic regression, variance inflation factor (VIF) values were examined; all VIFs were below 2.0, indicating acceptable levels of collinearity. Both variables were therefore retained in the final model.

Logistic regression was used to identify factors associated with membership in the more positive health belief profile. The Hosmer-Lemeshow goodness-of-fit test was used to assess model fit. The high-barrier/moderate-benefit profile was used as the reference category. Odds ratios (ORs), 95% confidence intervals (CIs) and *p* values were reported. A two-sided *p* value < 0.05 was considered statistically significant.

### 2.4 Ethics approval and consent to participate

The survey was anonymous, and participants were informed that their responses would be used only for academic research. Participation was voluntary. The study was approved by the Ethics Committee of Heze Municipal Hospital and Shenzhen Pingshan District Central Hospital (2024-KY003-06, PSZX-EC-AP-020). Anonymity was guaranteed, and data were used solely for research. The study was conducted in accordance with the Declaration of Helsinki. Informed consent was obtained from all participants before questionnaire completion. All methods were performed in accordance with the relevant guidelines and regulations (21 participants [0.8% of the total sample] were under 16 years of age; however, they had already been admitted to college through the national higher education entrance examination and were therefore considered to possess the cognitive capacity and legal competence to provide their own informed consent under Chinese law [Civil Code of the People’s Republic of China, Article 18]. The study protocol was reviewed and approved by the ethics committee for this specific context. All procedures were carried out in accordance with the relevant institutional and national guidelines.

## 3 Results

### 3.1 Descriptive statistics and reliability of main variables

The five health belief dimensions covered the full score range from 1 to 5, indicating heterogeneity in HPV vaccine-related health beliefs among college students. Perceived severity had the highest mean score (M = 3.802, SD = 0.919), followed by perceived benefits (M = 3.633, SD = 0.800) and self-efficacy (M = 3.463, SD = 0.787). Perceived barriers had a relatively lower mean score (M = 2.837, SD = 0.970). Cronbach’s alpha values were 0.836 for perceived susceptibility, 0.915 for perceived severity, 0.886 for perceived barriers, 0.880 for perceived benefits, 0.799 for self-efficacy, 0.862 for social support and perceived service accessibility, 0.969 for e-health literacy, and 0.916 for vaccination intention, indicating acceptable to excellent internal consistency (Table 1). HPV vaccine knowledge showed a KR-20 coefficient of 0.78, indicating acceptable reliability for the knowledge scale.

**Table 1:** Descriptive statistics and reliability of main variables.

Variable	Items	Mean	SD	Min	Max	Cronbach’s alpha
Perceived susceptibility	3	3.217	0.907	1.000	5.000	0.836
Perceived severity	3	3.802	0.919	1.000	5.000	0.915
Perceived barriers	3	2.837	0.970	1.000	5.000	0.886
Perceived benefits	3	3.633	0.800	1.000	5.000	0.880
Self-efficacy	2	3.463	0.787	1.000	5.000	0.799
Social support/accessibility	4	3.337	0.778	1.000	5.000	0.862
EHL (e-health literacy)	8	3.637	0.761	1.000	5.000	0.969
VI (vaccination intention)	4	3.571	0.762	1.000	5.000	0.916

SD, standard deviation; EHL, e-health literacy; VI, vaccination intention. All items were measured on a 5-point Likert scale ranging from 1 (strongly disagree) to 5 (strongly agree). HPV vaccine knowledge (not shown in this table) had a KR-20 coefficient of 0.78. Social support/accessibility refers to social support and perceived service accessibility.

### 3.2 Latent profile model fit

Models with two to six profiles were compared. The two-profile model had the lowest BIC (BIC = −29,142.8), acceptable entropy (0.802) and an adequate smallest class proportion (26.0%). The VLMR test was non-significant when comparing the three-profile against the two-profile solution (*p* = 0.067), indicating that the additional profile did not significantly improve model fit. Although the five- and six-profile models showed relatively high entropy, their smallest class proportions were only 4.5 and 3.9%, respectively, suggesting less stable and less interpretable small classes. Considering model fit, classification quality, class size and substantive interpretability, the two-profile model was selected as the final solution (Table 2). Although the BIC difference between the two- and three-profile solutions was small (ΔBIC = 6.3), the three-profile model had substantially lower entropy (0.569) and a less interpretable class structure, supporting retention of the more parsimonious two-profile solution.

**Table 2:** Model fit indices for latent profile analysis.

Number of profiles	BIC	Log-likelihood	Entropy	Class sizes	Smallest class (%)	Decision
2	−29,142.8	−14,428.8	0.802	2,040/715	26.0	Selected
3	−29,136.5	−14,362.3	0.569	1,449/477/829	17.3	
4	−28,500.0	−13,980.7	0.692	932/524/892/407	14.8	
5	−27,264.8	−13,299.7	0.853	124/275/1,026/988/342	4.5	
6	−26,578.0	−12,892.9	0.850	278/107/874/904/350/242	3.9	

BIC, Bayesian Information Criterion. The two-profile model was selected as the final solution based on BIC, entropy, class size, and theoretical interpretability.

### 3.3 Profile labels and characteristics

**Figure 1:**
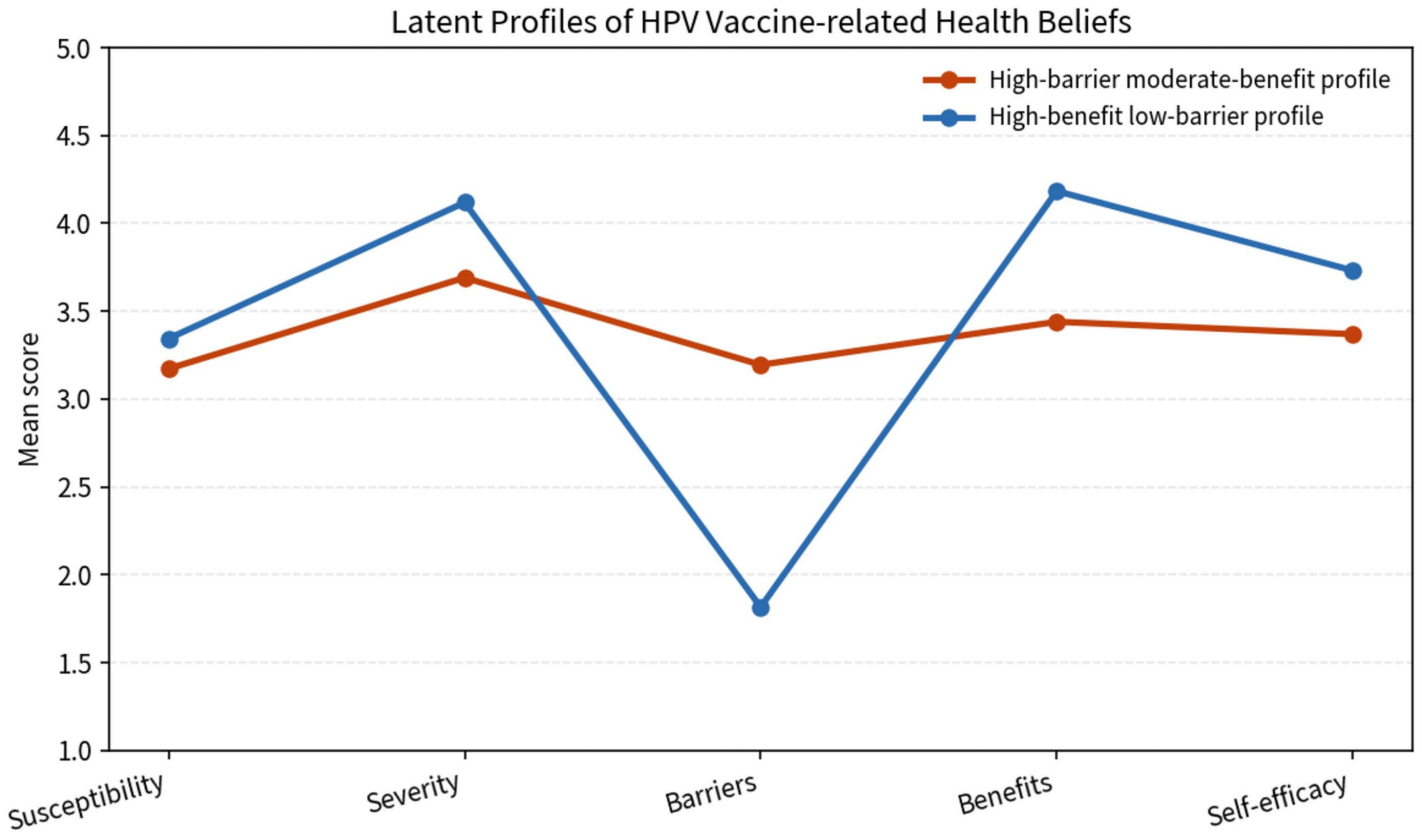
Latent profiles of HPV vaccine-related health beliefs.

Based on the mean patterns of the five health belief dimensions, the two latent profiles were labelled as the high-barrier/moderate-benefit profile and the high-benefit/low-barrier profile. The high-barrier/moderate-benefit profile included 2,040 participants (74.0%) and was characterized by relatively high perceived barriers and moderate levels of perceived benefits and self-efficacy. The high-benefit/low-barrier profile included 715 participants (26.0%) and was characterized by higher perceived benefits, perceived severity and self-efficacy, together with markedly lower perceived barriers. The two profiles differed significantly across all five health belief dimensions (Figure 1, Table 3).

**Table 3:** Differences in health belief dimensions between latent profiles.

Dimension	High-barrier/Moderate-benefit profile M (SD)	High-benefit/Low-barrier profile M (SD)	*t*	*p* value
Perceived susceptibility	3.17 (0.86)	3.34 (1.01)	−4.056	<0.001
Perceived severity	3.69 (0.91)	4.12 (0.88)	−11.062	<0.001
Perceived barriers	3.20 (0.82)	1.82 (0.56)	49.872	<0.001
Perceived benefits	3.44 (0.79)	4.18 (0.53)	−28.165	<0.001
Self-efficacy	3.37 (0.78)	3.73 (0.76)	−10.950	<0.001

Values are presented as mean (standard deviation). *p* values were derived from independent-samples *t* tests.

Figure 1 displays mean scores (with standard deviations) for the five Health Belief Model dimensions across the two latent profiles. Blue line: high-barrier/moderate-benefit profile (*n* = 2,040, 74.0%). Red line: high-benefit/low-barrier profile (*n* = 715, 26.0%). The high-barrier/moderate-benefit profile was characterized by relatively high perceived barriers and moderate levels of perceived benefits and self-efficacy. The high-benefit/low-barrier profile showed markedly lower perceived barriers and higher scores across all other dimensions, especially perceived benefits, perceived severity and self-efficacy. Error bars indicate standard deviations.

### 3.4 Sociodemographic and HPV-related characteristics across profiles

The two profiles differed in several sociodemographic and HPV-related characteristics. The proportion of female students was higher in the high-benefit/low-barrier profile than in the high-barrier/moderate-benefit profile (70.1% vs. 59.2%). Medical students were also more common in the high-benefit/low-barrier profile (34.7% vs. 24.6%). Significant differences were also observed in grade, age group, monthly living expenses and family economic status. In contrast, residence and age at first sexual intercourse did not differ significantly between profiles. Students in the high-benefit/low-barrier profile were more likely to have heard of HPV vaccine (95.7% vs. 86.1%), actively searched for HPV vaccine information (70.2% vs. 50.8%) and received HPV vaccination (24.6% vs. 15.3%; Table 4).

**Table 4:** Sociodemographic and HPV-related characteristics across latent profiles.

Variable	Category	High-barrier/Moderate-benefit *n* (%)	High-benefit/Low-barrier *n* (%)	*χ* ^2^	*p* value
Sex	Male	833 (40.8)	214 (29.9)	26.253	<0.001
	Female	1,207 (59.2)	501 (70.1)		
Discipline	Medical	501 (24.6)	248 (34.7)	26.917	<0.001
	Non-medical	1,539 (75.4)	467 (65.3)		
Grade	Freshman	944 (46.3)	369 (51.6)	11.871	0.018
	Sophomore	847 (41.5)	289 (40.4)		
	Junior	183 (9.0)	42 (5.9)		
	Senior	23 (1.1)	5 (0.7)		
	Other	43 (2.1)	10 (1.4)		
Age group	<18 years	20 (1.0)	1 (0.1)	36.133	<0.001
	18–21 years	127 (6.2)	11 (1.5)		
	22–26 years	1,742 (85.4)	666 (93.1)		
	27–30 years	142 (7.0)	36 (5.0)		
	Other	9 (0.4)	1 (0.1)		
Residence	Urban	436 (21.4)	172 (24.1)	2.063	0.151
	Rural	1,604 (78.6)	543 (75.9)		
Monthly living expenses	<800 yuan	221 (10.8)	34 (4.8)	27.826	<0.001
	800–1,200 yuan	720 (35.3)	252 (35.2)		
	1,201–1,800 yuan	869 (42.6)	324 (45.3)		
	1,801–2,500 yuan	189 (9.3)	90 (12.6)		
	>2,500 yuan	41 (2.0)	15 (2.1)		
Family economic status	Very affluent	39 (1.9)	2 (0.3)	32.404	<0.001
	Relatively affluent	122 (6.0)	61 (8.5)		
	Moderate	1,271 (62.3)	493 (69.0)		
	Relatively difficult	487 (23.9)	138 (19.3)		
	Very difficult	121 (5.9)	21 (2.9)		
Sexual debut	Never	1,674 (82.1)	604 (84.5)	5.928	0.205
	<16 years	23 (1.1)	4 (0.6)		
	16–23 years	130 (6.4)	49 (6.9)		
	>23 years	9 (0.4)	1 (0.1)		
	Prefer not to disclose	204 (10.0)	57 (8.0)		
Heard of HPV vaccine	No	283 (13.9)	31 (4.3)	46.744	<0.001
	Yes	1,757 (86.1)	684 (95.7)		
Actively searched HPV information	No	1,004 (49.2)	213 (29.8)	80.227	<0.001
	Yes	1,036 (50.8)	502 (70.2)		
Received HPV vaccine	No	1,728 (84.7)	539 (75.4)	30.924	<0.001
	Yes	312 (15.3)	176 (24.6)		

*n*, number of participants; %, percentage. *p* values were derived from chi-square tests. The “Other” category for grade included graduate students and non-standard academic tracks. The “Other” category for age group included participants aged >30 years.

### 3.5 Differences in knowledge, social support and perceived service accessibility, EHL, VI, and uptake

**Figure 2:**
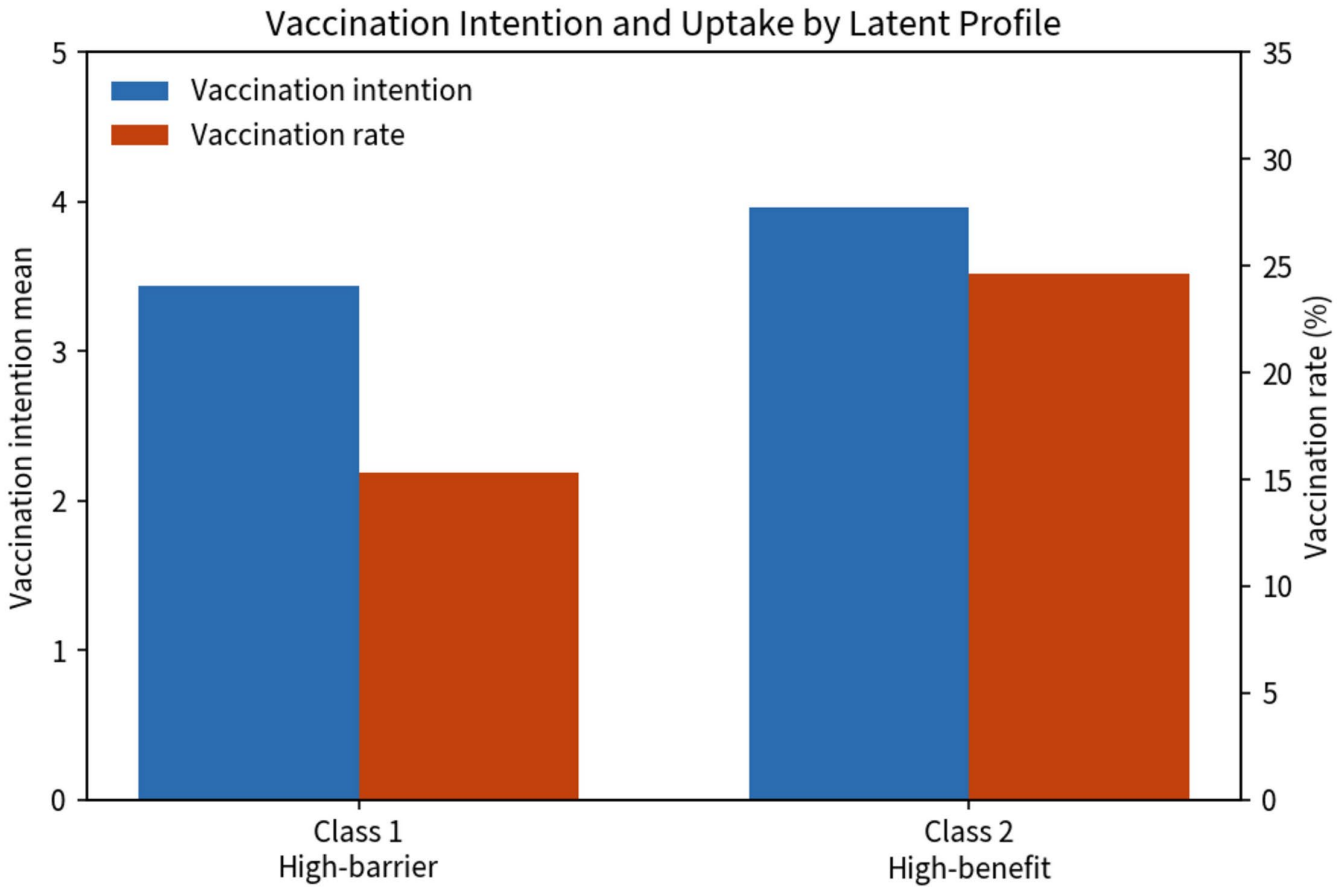
Vaccination intention and uptake by latent profile.

Students in the high-benefit/low-barrier profile had significantly higher HPV vaccine knowledge, social support and perceived service accessibility, EHL and VI than those in the high-barrier/moderate-benefit profile. The mean HPV vaccine knowledge score was 6.54 in the high-benefit/low-barrier profile, compared with 4.61 in the high-barrier/moderate-benefit profile. The mean vaccination intention score was also higher in the high-benefit/low-barrier profile (3.96 vs. 3.43). Effect size estimates (Cohen’s *d*) were 0.74 for knowledge, 0.33 for social support and perceived service accessibility, 0.69 for EHL, and 0.73 for vaccination intention, indicating small-to-medium effects for social support and medium-to-large effects for knowledge, EHL, and vaccination intention. For categorical variables, Cramér’s *V* values were 0.13 for having heard of HPV vaccine, 0.17 for actively searching HPV vaccine information, and 0.11 for receiving HPV vaccine, indicating small effects. The proportions of students who had heard of HPV vaccine, actively searched for HPV vaccine information and received HPV vaccination were all significantly higher in the high-benefit/low-barrier profile (Table 5, Figure 2).

**Table 5:** Differences in HPV vaccine-related knowledge, support, literacy, intention and uptake across profiles.

Variable	High-barrier/Moderate-benefit profile	High-benefit/Low-barrier profile	Statistic	*p* value	Effect size	Interpretation
HPV vaccine knowledge	4.61 (2.96)	6.54 (2.19)	−18.378	<0.001	Cohen’s *d* = 0.74	Medium-to-large difference
Social support/accessibility	3.26 (0.76)	3.55 (0.78)	−8.675	<0.001	Cohen’s *d* = 0.33	Small-to-medium difference
EHL	3.51 (0.75)	4.00 (0.68)	−16.385	<0.001	Cohen’s *d* = 0.69	Medium-to-large difference
VI	3.43 (0.73)	3.96 (0.71)	−17.048	<0.001	Cohen’s *d* = 0.73	Medium-to-large difference
Heard of HPV vaccine	1,757 (86.1)	684 (95.7)	46.744	<0.001	Cramer’s *V* = 0.13	Small effect
Actively searched HPV information	1,036 (50.8)	502 (70.2)	80.227	<0.001	Cramer’s *V* = 0.17	Small effect
Received HPV vaccine	312 (15.3)	176 (24.6)	30.924	<0.001	Cramer’s *V* = 0.11	Small effect

EHL, e-health literacy; VI, vaccination intention. Values are presented as mean (standard deviation) for continuous variables and *n* (%) for categorical variables. *p* values were derived from independent-samples *t* tests for continuous variables and chi-square tests for categorical variables.

Figure 2 displays vaccination intention scores (bars, left y-axis) and vaccination uptake rates (dots with 95% CI, right y-axis) for the two latent profiles. Between-profile differences in both vaccination intention and uptake were statistically significant (*p* < 0.001).

### 3.6 Factors associated with latent profile membership

Using the high-barrier/moderate-benefit profile as the reference group, logistic regression was conducted to identify factors associated with membership in the high-benefit/low-barrier profile. The Hosmer-Lemeshow goodness-of-fit test indicated acceptable model fit (*χ*^2^ = 8.14, df = 8, *p* = 0.420), and the Nagelkerke *R*^2^ was 0.187. Female students had greater odds of belonging to the high-benefit/low-barrier profile (OR = 1.268, 95% CI: 1.03–1.56, *p* = 0.026). Higher grade was associated with lower odds of belonging to this profile (OR = 0.815, 95% CI: 0.71–0.93, *p* = 0.002). Active information seeking was positively associated with membership in the high-benefit/low-barrier profile (OR = 1.340, 95% CI: 1.09–1.65, *p* = 0.005). Higher HPV vaccine knowledge (OR = 1.222, 95% CI: 1.17–1.28, *p* < 0.001) and higher EHL (OR = 2.334, 95% CI: 1.98–2.76, *p* < 0.001) were also associated with greater odds of belonging to the high-benefit/low-barrier profile. Among all predictors, EHL exhibited the strongest association with profile membership (OR = 2.334). Social support and perceived service accessibility showed a negative association with membership in the high-benefit/low-barrier profile after adjustment for knowledge and EHL (OR = 0.843, 95% CI: 0.73–0.98, *p* = 0.024). This finding is considered further in the Discussion (Table 6).

**Table 6:** Logistic regression analysis of factors associated with membership in the high-benefit/low-barrier profile.

Predictor	OR	95% CI	*p* value
Female	1.268	1.03–1.56	0.026
Medical discipline	1.225	1.00–1.51	0.055
Grade	0.815	0.71–0.93	0.002
Age group	1.144	0.85–1.55	0.383
Urban residence	1.037	0.82–1.31	0.758
Monthly living expenses	1.088	0.96–1.24	0.198
Family economic status	0.948	0.82–1.10	0.481
Ever had sex	1.139	0.80–1.63	0.473
Heard of HPV vaccine	1.464	0.96–2.23	0.077
Actively searched HPV information	1.340	1.09–1.65	0.005
HPV vaccine knowledge	1.222	1.17–1.28	<0.001
Social support/accessibility	0.843	0.73–0.98	0.024
EHL	2.334	1.98–2.76	<0.001

OR, odds ratio; CI, confidence interval. The high-barrier/moderate-benefit profile served as the reference category. Variables were entered simultaneously into the regression model. *p* values < 0.05 were considered statistically significant.

## 4 Discussion

To our knowledge, this is among the first studies to use LPA to examine HPV vaccine-related health belief profiles among Chinese college students within the HBM framework. The person-centered approach revealed two distinct belief profiles: a high-barrier/moderate-benefit profile (74.0%) and a high-benefit/low-barrier profile (26.0%). From an interpretive perspective, the high-barrier/moderate-benefit profile may be understood as a group of students who recognized some preventive value of HPV vaccination but remained constrained by perceived barriers. This interpretation should be viewed as a summary of the observed belief pattern rather than an independently measured psychological category. The high-benefit/low-barrier profile can be characterized as students who perceived strong benefits and low barriers, possessed higher knowledge and e-health literacy, and were more proactive in information seeking.

### 4.1 The high-barrier/moderate-benefit majority: implications for intervention

The predominance of the high-barrier/moderate-benefit profile has important implications for the promotion of HPV vaccination. The findings of this study indicate that the absence of VI among college students cannot be solely attributed to a deficiency in awareness or knowledge. While many students may already have some recognition of the vaccine’s preventive value, perceived barriers may prevent this recognition from being translated into VI and uptake (30, 31). Interventions should not be limited to the dissemination of general knowledge. It is imperative that health education for students in this profile directly addresses concerns regarding vaccination. This should be achieved by providing clear, credible and practical information about vaccine safety, common adverse reactions, vaccination cost, appointment procedures and service accessibility (32). For this group, health education strategies may need to combine barrier-specific communication with practical access support, including transparent information about vaccine cost, evidence-based communication about vaccine safety and adverse reactions, guidance on appointment procedures, and improved access to vaccination services. From a health education perspective, this group would benefit most from interventions that combine risk communication with practical barrier-reduction strategies, delivered through channels that students trust and access regularly.

### 4.2 The high-benefit/low-barrier minority: from intention to action

Students who exhibited a high-benefit/low-barrier profile demonstrated a more positive health belief pattern. The study reported higher perceived benefits, perceived severity and self-efficacy, and markedly lower perceived barriers. Furthermore, the study found that the subjects had a higher level of knowledge regarding the HPV vaccine, EHL, social support and perceived service accessibility, VI and vaccination uptake. For this group, interventions may focus less on basic awareness-raising and more on facilitating action, such as providing appointment information, vaccination site availability, vaccine type options and reminders for timely vaccination. The difference between vaccination intention and uptake may indicate that favorable beliefs do not automatically translate into vaccination behavior. However, because vaccination behavior was measured cross-sectionally, this interpretation should be considered provisional. For this group, health promotion efforts may place greater emphasis on behavioral facilitation, removing practical barriers to access through convenient on-campus vaccination services, streamlined appointment systems, and follow-up reminders, rather than further knowledge dissemination.

### 4.3 E-health literacy as the strongest predictor

The strong association between EHL and membership in the high-benefit/low-barrier profile is noteworthy. In the current digital age, college students often get vaccine information from online platforms and social media (33, 34). Students with higher EHL may be better able to identify credible sources and evaluate conflicting online vaccine information. However, this mechanism was not directly tested in the present study and should be examined in future research. This finding is especially important for middle-income countries like China, where more and more people are using the internet to find health information, but not everyone can effectively evaluate it. From a health education and promotion standpoint, EHL represents a potentially modifiable target for intervention. Universities may consider integrating EHL training into existing health education curricula, teaching students how to critically evaluate online health information, identify credible sources, and make informed vaccination decisions. This approach aligns with the growing emphasis on digital health literacy as a core competency for 21st-century health promotion (35).

### 4.4 Gender disparities in vaccine beliefs

Female college students were more likely to belong to the high-benefit/low-barrier profile. This may be because HPV vaccines are often promoted for preventing cervical cancer, which makes female students more aware of the importance of HPV vaccines (16, 25). HPV infection and HPV-related diseases also affect men, and men may contribute to HPV transmission (36). HPV vaccination promotion on college campuses should not be limited to women. Male students may require targeted education emphasizing the benefits of HPV vaccination for their own health, partner protection and population-level prevention. This gender disparity in vaccine beliefs is consistent with findings from other countries, highlighting a global challenge that requires attention in vaccination promotion efforts (37, 38). It is recommended that future interventions adopt a gender-inclusive framing of HPV vaccination as a universal cancer prevention strategy rather than a women’s health issue. Health education campaigns should therefore be designed with gender-sensitive messaging that resonates with both male and female students, emphasizing shared responsibility for cancer prevention.

### 4.5 Active information seeking and its role

Active information seeking was also associated with membership in the high-benefit/low-barrier profile. Students who proactively seek information regarding the HPV vaccine may possess a more comprehensive understanding of its benefits and address any existing uncertainties (39, 40). However, this hypothesized mechanism should be examined in future research. Health promotion programs should encourage active information seeking while guiding students toward credible information sources (41, 42). In order to reduce reliance on fragmented or inaccurate information, universities may provide curated online resources, campus health service platforms or professional consultation channels (43). This finding underscores the importance of health education strategies that not only provide accurate information but also foster students’ motivation and skills to actively seek, evaluate, and apply health information—a core objective of health literacy promotion.

### 4.6 The complex role of social support and perceived service accessibility

The negative adjusted association between social support and perceived service accessibility and membership in the high-benefit/low-barrier profile should be interpreted cautiously. Because this variable was higher in the favorable profile in bivariate comparisons, the reversed direction in the multivariable model may reflect shared variance with HPV vaccine knowledge and e-health literacy or a statistical suppression effect. Another possible explanation is that social support and perceived service accessibility may differ in quality and source; support from peers or family members with inaccurate vaccine information may not necessarily promote favorable beliefs (39). However, these explanations are hypothetical and should be examined in future studies using measures that distinguish informational, emotional, professional, and service-accessibility components of support (44, 45). For health educators, this finding suggests that interventions should consider the quality and source of support, not just its presence. Peer education programs that train students to provide accurate vaccine information may help transform social networks from potential barriers into facilitators of positive health beliefs, but their effectiveness should be tested in future longitudinal or intervention studies.

### 4.7 Global health implications and transferability

China has a distinctive HPV vaccination context, including relatively recent vaccine introduction, self-paid vaccination for many students, regional differences in access, and specific cultural meanings surrounding sexual and reproductive health (46). Therefore, the prevalence and characteristics of the profiles identified in this study should not be assumed to apply directly to other countries. Nevertheless, the person-centered method used in this study may be informative for HPV vaccination research in other settings. Latent profile analysis has been applied in vaccine attitude and vaccine hesitancy research in other populations, showing its value for identifying subgroups with distinct belief or attitude patterns (47). The present study extends this approach to HPV vaccine-related health beliefs among Chinese college students. Future studies in other countries are needed to examine whether similar or different profiles emerge under different healthcare systems, cultural contexts, and vaccination policies.

### 4.8 Strengths and limitations

This study has several strengths. First, it was grounded in the HBM and used a person-centered approach to identify heterogeneous HPV vaccine-related belief patterns among college students. Second, the large sample size enabled reliable identification of distinct profiles. Third, the study examined profile characteristics as well as differences in HPV vaccine knowledge, social support and perceived service accessibility, e-health literacy, vaccination intention, and uptake, providing evidence for more differentiated health education strategies. Fourth, the study contributes empirical evidence from China, a context that remains underrepresented in international research on HPV vaccine-related belief profiles.

Several limitations should be acknowledged. First, the cross-sectional design does not allow causal inference. Associations between profile membership, vaccination intention and uptake should be interpreted as correlational. Second, all variables were self-reported and may be affected by recall bias or social desirability bias. Third, although the two-profile solution was statistically and theoretically interpretable, profile stability should be further validated in independent samples. Fourth, although the two-profile model was selected based on BIC, entropy, and interpretability, the BIC difference between the two- and three-profile solutions was relatively small. The VLMR test provided additional support, but the stability and generalizability of the two-profile solution should be confirmed in future studies with larger and more diverse samples. Fifth, vaccination uptake was measured as receipt of at least one dose, and information on vaccine type, number of doses completed, and vaccination timing was not available. Sixth, the study used mean scores for LPA at the dimension level; future research may examine how patterns or changes over time show up at the item level or in health belief profiles over time. Seventh, participants were recruited using convenience sampling from five universities in two provinces, which may limit the representativeness of the sample and the generalizability of the findings to all Chinese college students. Eighth, although multiple sociodemographic and HPV-related variables were included in the regression model, residual confounding from unmeasured factors, such as parental attitudes, local vaccine supply, prior healthcare experiences, and exposure to vaccine-related misinformation, cannot be excluded. Ninth, the latent profiles were identified in a single sample and were not externally validated. Future studies should examine whether the two-profile structure can be replicated in independent samples from different regions and institutional contexts.

## 5 Conclusion

This study identified two distinct HPV vaccine-related health belief profiles among Chinese college students: a high-barrier/moderate-benefit profile and a high-benefit/low-barrier profile. The predominance of the high-barrier/moderate-benefit profile indicates that many students recognized some preventive value of HPV vaccination while still reporting substantial concerns related to cost, safety, adverse reactions, vaccine effectiveness, and access to vaccination services. By applying latent profile analysis to Health Belief Model-based dimensions, this study provides a person-centered understanding of HPV vaccine-related beliefs and complements previous variable-centered studies of vaccination intention and uptake.

Female students, active information seeking, higher HPV vaccine knowledge, and higher e-health literacy were associated with membership in the high-benefit/low-barrier profile. Among these factors, e-health literacy showed the strongest adjusted association in the multivariable model. However, because this study was cross-sectional, these findings should be interpreted as associations rather than causal effects. HPV vaccine knowledge, active information seeking, and e-health literacy may represent potential targets for future intervention studies, but longitudinal and experimental research is needed to determine whether improving these factors leads to more favorable beliefs or higher vaccination uptake.

The person-centered profiling approach used in this study may offer a useful methodological reference for future HPV vaccination research in other settings. However, its applicability should be evaluated in light of differences in healthcare systems, vaccination policies, cultural norms, vaccine financing mechanisms, and vaccine accessibility. Future studies should validate the identified profiles in independent and more diverse samples and examine whether profile-specific health education strategies are effective in improving HPV vaccination decision-making and uptake.

## Data Availability

The original contributions presented in the study are included in the article/Supplementary material, further inquiries can be directed to the corresponding authors.

## References

[1] Jensen JE Becker GL Jackson JB Rysavy MB. Human papillomavirus and associated cancers: a review. Viruses. (2024) 16:680. doi: 10.3390/v16050680, 38793561 PMC11125882

[2] Shapiro GK. HPV vaccination: an underused strategy for the prevention of cancer. Curr Oncol. (2022) 29:3780–92. doi: 10.3390/curroncol29050303, 35621693 PMC9140027

[3] World Health Organization. Global Strategy to Accelerate the Elimination of Cervical Cancer as a Public Health Problem. Geneva: World Health Organization (2020).

[4] Defo VF Domgue JF. Understanding the WHO global strategy to accelerate cervical cancer elimination. Lancet Glob Health. (2026) 14:e333–4. doi: 10.1016/S2214-109X(26)00004-5 41554270

[5] World Health Organization. Human papillomavirus vaccines: WHO position paper. Wkly Epidemiol Rec. (2022) 97:645–72.

[6] Sophian A Normasari Harumanto A Abinawanto. Global vaccine confidence at a crossroads: immunisation coverage declines, misinformation, and the future of vaccine policy. Rev Med Virol. (2026) 36:e70149. doi: 10.1002/rmv.70149 41999127

[7] Guo L Guo F Jiang W Zhang X Dong D Chen S . Availability of Hib, pneumococcal, rotavirus, and HPV vaccines in China: implication for equity in access to immunization services. PLoS Glob Public Health. (2026) 6:e0006151. doi: 10.1371/journal.pgph.0006151, 42013154 PMC13098980

[8] Song S-Y Guo Y Li Y-H Wang Z Gao W. Analysis of factors influencing HPV vaccination intention among Chinese college students: structural equation modeling based on health belief theory. Front Public Health. (2025) 12:1510193. doi: 10.3389/fpubh.2024.1510193, 39949340 PMC11821927

[9] Xu Y Wang W Cheng C Yang L Xing C Yang X . Factors influencing HPV vaccination willingness among male college students in Jinan according to the health belief model. Sci Rep. (2025) 15:30369. doi: 10.1038/s41598-025-16299-5, 40830565 PMC12365129

[10] Zhuang Y Kwang Cheol K Botabara-Yap MJ Zhao K Ramos RIA Cao W. Factors associated with reproductive health and health education participation among female college students in China. Front Public Health. (2025) 13:1627669. doi: 10.3389/fpubh.2025.1627669, 40959616 PMC12433993

[11] Malik S Mock KO Martillotti R Caravella G Zhou X Mbamelu M . HPV vaccines among university students: understanding barriers and facilitators of vaccine uptake. Vaccine. (2024) 12:1385. doi: 10.3390/vaccines12121385, 39772047 PMC11680171

[12] Çevik HS Amariutei A Mazur A Peker GC Görpelioğlu S Vinker S . Unlocking the key to HPV prevention: exploring factors influencing HPV vaccination decisions among young people and their parents. Public Health. (2025) 238:214–20. doi: 10.1016/j.puhe.2024.12.016, 39689649

[13] Dapari R Li M Chen X Cui J Zamzuri MAIA Hassan MR . Factors influencing HPV vaccine acceptance among females in mainland China: a systematic review. Clin Epidemiol Glob Health. (2024) 26:101514. doi: 10.1016/j.cegh.2024.101514

[14] Mohammadi S Rabiei Z Pajohideh ZS Barati Z Talebi SS Keramat A. Evaluating the health belief model constructs in adopting the HPV preventive behavior. J Family Reprod Health. (2023) 17:37–44. doi: 10.18502/jfrh.v17i1.11975, 37538224 PMC10394485

[15] Abuzoor A Jabin MSR Eshareturi C Nesbitt R Jonker C. Using the health belief model to examine parental knowledge and health beliefs about human papilloma virus (HPV) and iHPV vaccine in Kuwait: cross-sectional survey study. JMIR Public Health Surveill. (2025) 11:e75818. doi: 10.2196/75818, 41368717 PMC12690283

[16] Houtin L Romo L Joret Y Brun C. Perceptions of HPV vaccination: a qualitative study with adolescents, parents, school staff, and pharmacists. BMC Med. (2026) 24. doi: 10.1186/s12916-026-04821-z, 41888784 PMC13141285

[17] Wu Y Fu Y Zuo C Chen C Zhang L Chen T. Gender differences in HPV vaccine hesitancy among college students: implications for targeted health education strategies. Vaccine. (2026) 79:128471. doi: 10.1016/j.vaccine.2026.128471, 41830691

[18] Tobaiqy M MacLure K. A systematic review of human papillomavirus vaccination challenges and strategies to enhance uptake. Vaccine. (2024) 12:746. doi: 10.3390/vaccines12070746, 39066384 PMC11281456

[19] Li Q Fang F Zhang Y Tu J Zhu P Xi L. eHealth literacy and its outcomes among postsecondary students: systematic review. J Med Internet Res. (2025) 27:e64489. doi: 10.2196/64489, 40601925 PMC12278882

[20] Ibrahim AM Zaghamir DEF. Impact of health education on knowledge and sexually transmitted infections prevention in young married women: quasi-experimental study. Public Health Nurs. (2026) 43:1056–66. doi: 10.1111/phn.70093, 41782216

[21] Moore EWG Quartiroli A. Representing subpopulations with latent profile analysis: a non-technical introduction using exercisers’ goal orientation adoption profiles. J Behav Med. (2025) 49:123–37. doi: 10.1007/s10865-025-00596-5, 40924355 PMC13253718

[22] Spurk D Hirschi A Wang M Valero D Kauffeld S. Latent profile analysis: a review and “how to” guide of its application within vocational behavior research. J Vocat Behav. (2020) 120:103445. doi: 10.1016/j.jvb.2020.103445

[23] Mancuso N Michaels J Browne EN Maragh-Bass AC Stocks JB Soberano ZR . Greater improvements in vaccination outcomes among black young adults with vaccine-resistant attitudes in the United States south following a digital health intervention: latent profile analysis of a randomized control trial. JMIR Public Health Surveill. (2025) 11:e67370–13. doi: 10.2196/67370, 40239211 PMC12017611

[24] Fields EJ. Vaccine Acceptance Among African American and Black Adults in South Central Los Angeles: A Latent Profile Analysis Study.University of California, Irvine (2025).

[25] Li W Jin Y Li X Jin M. Human papillomavirus and vaccine knowledge, willingness, and uptake among university students: a systematic review and meta-analysis. BMC Public Health. (2025) 25:4268. doi: 10.1186/s12889-025-25626-4, 41419843 PMC12717715

[26] Xie H Zhou M Zhang J He J Bai Z Zuo Y. Urban–rural disparities in HPV vaccination knowledge, willingness, and uptake among women in the Tibet autonomous region: a multi-center cross-sectional study. Front Public Health. (2026) 14:1796545. doi: 10.3389/fpubh.2026.1796545, 42305742 PMC13265519

[27] Jin J Han Y. Understanding cultural perceptions of sexuality in China and their influence on human papillomavirus vaccine hesitancy. Front Public Health. (2025) 12:1462722. doi: 10.3389/fpubh.2024.1462722, 39917522 PMC11801254

[28] Xu Y Pierce JD Anderson K Dawkins-Moultin L Cho D Hopfer S . From knowledge to action: effects of HPV knowledge, HPV vaccine attitude, gender, and HPV vaccine safety concerns on HPV vaccination intent. J Health Commun. (2026) 31:90–100. doi: 10.1080/10810730.2026.2624671, 41729572

[29] Norman CD Skinner HA. eHEALS: the eHealth literacy scale. J Med Internet Res. (2006) 8:e27. doi: 10.2196/jmir.8.4.e27, 17213046 PMC1794004

[30] Wang W. The impact of vaccine access difficulties on HPV vaccine intention and uptake among female university students in China. Int J Equity Health. (2025) 24:4. doi: 10.1186/s12939-024-02370-6, 39780149 PMC11715876

[31] Alnezary FS Alhejaili RA Alayashi AA Alnizari DS Bin Mirdhah NS Alrays SK . Risk perception and influenza vaccine hesitancy among university students in Saudi Arabia. Front Pharmacol. (2026) 17:1747976. doi: 10.3389/fphar.2026.1747976, 41635482 PMC12862249

[32] Chen X Xia S Zhu Z Hui Z Wu J Sun C . Influenza vaccine hesitancy in older adults in China: a latent profile analysis. Hum Vaccin Immunother. (2026) 22:2616943. doi: 10.1080/21645515.2026.2616943, 41689585 PMC12915807

[33] Apata BO Tupe AH Akeju O Wilson KL. Impact of social media on HPV vaccine knowledge and attitudes among adolescents and young adults: a systematic literature review. Healthcare (Basel). (2025) 14:73. doi: 10.3390/healthcare14010073, 41517004 PMC12785451

[34] Nichol B Erfani G McCready J Gordon C Unsworth J Croston M . The impact of social media addiction on vaccine hesitancy among nursing students: testing an empirical model. Nurs Open. (2026) 13:e70460. doi: 10.1002/nop2.70460, 41840357 PMC13097686

[35] Ammar N Olusanya OA Melton C Chinthala L Huang X White BM . Digital personal health coaching platform for promoting human papillomavirus infection vaccinations and cancer prevention: knowledge graph-based recommendation system. JMIR Form Res. (2023) 7:e50210. doi: 10.2196/50210, 37966885 PMC10687687

[36] Beltrame A Dube Mandishora RS Wang W Fan W Haver MK Giuliano AR. Impact of male human papillomavirus (HPV) vaccination on HPV infection and HPV-related diseases: a systematic review. Rev Med Virol. (2026) 36:e70171. doi: 10.1002/rmv.70171, 42217245

[37] Nyasulu BJ Heidari S Manna M Bahl J Goodman T. Gender analysis of the World Health Organization online learning program on immunization agenda 2030. Front Glob Women's Health. (2023) 4:1230109. doi: 10.3389/fgwh.2023.1230109, 38152380 PMC10751919

[38] Sad SA Iftikhar L Chamout M. Revisiting HPV vaccination post-COVID: geopolitical, sociocultural, and ethical disparities in global health. Int J Equity Health. (2025) 24:308. doi: 10.1186/s12939-025-02669-y, 41214693 PMC12604161

[39] Giannella L Grelloni C Natalini L Sartini G Lavezzo F Cicoli C . The role of internet information on anti-HPV vaccines: a comprehensive overview of a double-edged sword. Vaccine. (2025) 13:445. doi: 10.3390/vaccines13050445, 40432056 PMC12116087

[40] Wang T-CL Zhang M-J Zhang H. Examining the impact of sex-biased information on health behaviors: a study of HPV vaccination among male college students based on the extended theory of planned behavior. Front Public Health. (2025) 13:1525547. doi: 10.3389/fpubh.2025.1525547, 40416654 PMC12098300

[41] Vader AM Walters ST Roudsari B Nguyen N. Where do college students get health information? Believability and use of health information sources. Health Promot Pract. (2011) 12:713–22. doi: 10.1177/1524839910369995, 21282492

[42] Minghao P Leyun H Jingying Y Wanyu H Fan W Linlin W . A grounded theory study on medical students’ proxy online health information seeking behavior. BMC Public Health. (2025) 25:339. doi: 10.1186/s12889-025-21394-3, 39871230 PMC11773963

[43] Mahama IC Katuuk DA Umbase R Tambingon HN. Social media use, digital literacy, and counseling effectiveness as predictors of reproductive health knowledge among medical students: an educational management perspective. Int. J. Inf. Technol. Educ. (2026) 5:176–92. https://ijite.jredu.id/index.php/ijite/article/view/367/315

[44] Rosário J Pires JC Dias S Pedro AR. Exploring perceptions of health literacy, healthcare access, and utilisation among higher education students in Alentejo, southern Portugal: a qualitative study. PLoS One. (2025) 20:e0326575. doi: 10.1371/journal.pone.0326575, 40577325 PMC12204513

[45] Kong H Geng J Wang Q Zhu J Zhao H. Exploring the mediating roles of stigma and perceived social support between health literacy and health care decision-making barriers among patients with cervical cancer. Asia Pac J Oncol Nurs. (2025) 12:100716. doi: 10.1016/j.apjon.2025.100716, 40503356 PMC12152865

[46] Wang L Zhong Y Di J. Current experience in HPV vaccination in China. Indian J Gynecol Oncol. (2021) 19:50. doi: 10.1007/s40944-021-00535-7

[47] Choi Y Leung K Wu JT Larson HJ Lin L. Identifying vaccine-hesitant subgroups in the Western Pacific using latent class analysis. npj Vaccines. (2025) 10:29. doi: 10.1038/s41541-025-01067-3, 39939318 PMC11821871

