# Supplementary material for: Latent profiles of HPV vaccine-related health beliefs and their associations with vaccination intention among college students: a global health perspective
Source: Front Public Health. 2026 Jul 28;14:1918113. doi: 10.3389/fpubh.2026.1918113 (PMC13457725; doi:10.3389/fpubh.2026.1918113)
Supplement: Supplementary file 1 [file Data_sheet_1.docx]

**Supplementary Materials**

**Latent profiles of HPV vaccine-related health beliefs and their associations with vaccination intention among college students: A Global Health Perspective**

Note. This supplementary file includes the full questionnaire, variable coding and scoring rules, latent profile model fit indices, effect size estimates, reviewer-response revision checklist, and two supplementary figures. Tables and figures are numbered consecutively as supplementary materials.

# Supplementary Table 1. Full Questionnaire

## Section A. Sociodemographic and HPV-Related Characteristics

| **No.** | **Item** | **Response Options** |
| --- | --- | --- |
| A1 | Sex | A. Male; B. Female |
| A2 | Discipline | A. Medical; B. Non-medical |
| A3 | Grade | A. Freshman; B. Sophomore; C. Junior; D. Senior; E. Other |
| A4 | Age group | A. <18 years; B. 18-21 years; C. 22-26 years; D. 27-30 years; E. Othe |
| A5 | Residence | A. Urban; B. Rural |
| A6 | Monthly living expenses | A. <800 yuan; B. 800-1200 yuan; C. 1201-1800 yuan; D. 1801-2500 yuan; E. >2500 yuan |
| A7 | Family economic status | A. Very affluent; B. Relatively affluent; C. Moderate; D. Relatively difficult; E. Very difficult |
| A8 | Age at first sexual intercourse | A. Never; B. <16 years; C. 16-23 years; D. >23 years; E. Prefer not to disclose |
| A9 | Have you heard of HPV vaccine? | A. Yes; B. No |
| A10 | Have you received HPV vaccine? | A. Yes, at least one dose; B. No |
| A11 | Main source of HPV vaccine knowledge | A. Community/school health education; B. Television/internet; C. Healthcare professionals; D. Family/friends; E. No source or do not know |
| A12 | Have you actively searched for HPV vaccine-related information? | A. Yes; B. No |

## Section B. HPV Vaccine Knowledge

Knowledge items were scored as 1 for correct answers and 0 for incorrect or uncertain answers. Total scores ranged from 0 to 9, with higher scores indicating greater HPV vaccine knowledge.

| **No.** | **Item** |
| --- | --- |
| K1 | HPV vaccine can be administered from the age of 9 years. |
| K2 | HPV vaccine cannot be administered after the age of 45 years. |
| K3 | HPV vaccination can effectively prevent cervical cancer. |
| K4 | Both males and females can receive HPV vaccination. |
| K5 | HPV screening is no longer needed after HPV vaccination. |
| K6 | The nine-valent HPV vaccine covers the largest number of HPV types. |
| K7 | HPV vaccination is best administered before first sexual intercourse. |
| K8 | Individuals who have had sexual intercourse can still receive HPV vaccination. |
| K9 | HPV vaccine can treat existing HPV infection. |

## Section C. HPV Vaccine-Related Health Beliefs

All items were measured on a 5-point Likert scale: 1 = strongly disagree, 2 = disagree, 3 = neutral, 4 = agree, 5 = strongly agree.

| **Dimension** | **No.** | **Item** |
| --- | --- | --- |
| Perceived susceptibility | HB1 | If I do not receive HPV vaccination, I may be at risk of HPV infection or cervical cancer in the future. |
| Perceived susceptibility | HB2 | If I do not receive HPV vaccination, I may be at risk of sexually transmitted diseases in the future. |
| Perceived susceptibility | HB3 | If I do not undergo regular cervical cancer screening, my risk of HPV infection may increase in the future. |
| Perceived severity | HB4 | HPV infection would be a serious health problem for me. |
| Perceived severity | HB5 | HPV infection would affect my physical health, work, or marriage. |
| Perceived severity | HB6 | Cervical cancer would increase my psychological concerns and family economic burden. |
| Perceived barriers | HB7 | The high cost of HPV vaccination would prevent me from receiving the vaccine. |
| Perceived barriers | HB8 | Concerns about the safety and effectiveness of HPV vaccine would prevent me from receiving the vaccine. |
| Perceived barriers | HB9 | Concerns about adverse reactions after vaccination, such as pain, swelling, or nausea, would prevent me from receiving HPV vaccine. |
| Perceived benefits | HB10 | HPV vaccination can help reduce the risk of HPV infection. |
| Perceived benefits | HB11 | HPV vaccination can help prevent cervical cancer and other cancers, such as anal cancer. |
| Perceived benefits | HB12 | HPV vaccination can help reduce the risk of genital warts. |
| Self-efficacy | HB13 | I believe that the protective effect of HPV vaccination can last for a long time. |
| Self-efficacy | HB14 | I believe that HPV vaccination is more efficient than other preventive methods, such as maintaining hygiene or regular screening. |

## Section D. HPV Vaccination Intention and Related Behavioral Items

| **No.** | **Item** | **Response Options** |
| --- | --- | --- |
| VI1 | I would consult about HPV vaccination through social media. | 1-5 Likert scale |
| VI2 | I plan to receive HPV vaccination. | 1-5 Likert scale |
| VI3 | I believe that I will receive HPV vaccination. | 1-5 Likert scale |
| VI4 | I would recommend HPV vaccination to friends or relatives. | 1-5 Likert scale |
| B1 | What is the maximum acceptable price for three doses of HPV vaccine? | A. <600 yuan; B. 601-1000 yuan; C. 1001-2000 yuan; D. 2001-5000 yuan; E. >5000 yuan |
| B2 | Reasons for not receiving HPV vaccination | Multiple choice: A. Have not heard of it or do not understand it; B. Fear of side effects or adverse reactions; C. High price; D. Concern about social misunderstanding related to sexual behavior; E. Believe I am not at risk; F. Do not know where to receive vaccination; G. Other |

## Section E. Social Support and Perceived Service Accessibility

| **No.** | **Item** |
| --- | --- |
| SS1 | My parents or family members encourage me to receive HPV vaccination and are willing to accompany me to the hospital. |
| SS2 | Some classmates or friends have actively recommended HPV vaccination to me and shared their vaccination experiences. |
| SS3 | School doctors or other healthcare professionals have advised me to receive HPV vaccination. |
| SS4 | HPV vaccination can be easily scheduled at community health service centers in my area. |

## Section F. E-Health Literacy

| **No.** | **Item** |
| --- | --- |
| EHL1 | I know how to find useful health information online. |
| EHL2 | I know how to use the internet to answer my health questions. |
| EHL3 | I know what health resources are available online. |
| EHL4 | I know where to find useful health resources online. |
| EHL5 | I know how to use online health information to help myself. |
| EHL6 | I have the skills to evaluate the quality of online health information. |
| EHL7 | I can distinguish high-quality from low-quality online health information. |
| EHL8 | I feel confident in using online information to make health-related decisions. |

# Supplementary Table 2. Variable Coding and Scoring Rules

| **Construct/Variable** | **Items** | **Coding/Scoring** | **Score Range** | **Interpretation** |
| --- | --- | --- | --- | --- |
| Sex | A1 | Male = 0; Female = 1 | 0-1 | Binary sociodemographic variable |
| Discipline | A2 | Non-medical = 0; Medical = 1 | 0-1 | Binary academic background variable |
| Grade | A3 | Year 1-5 coded ordinally | 1-5 | Higher values indicate higher grade level |
| Age group | A4 | Categories retained as ordinal age groups | 1-5 | Higher values indicate older age group |
| Residence | A5 | Rural = 0; Urban = 1 | 0-1 | Binary residence variable |
| Monthly living expenses | A6 | Ordered from lowest to highest category | 1-5 | Higher values indicate higher monthly living expenses |
| Family economic status | A7 | Ordered from very difficult to very affluent or retained as categories according to analysis | 1-5 | Higher values indicate better family economic status if recoded directionally |
| Age at first sexual intercourse | A8 | <18, 18-21, 22-26, 27-30, Other | Categorical | Used as categorical variable; may be recoded as ever had sex vs. never/prefer not to disclose when appropriate |
| Heard of HPV vaccine | A9 | No = 0; Yes = 1 | 0-1 | HPV vaccine awareness |
| Received HPV vaccine | A10 | No = 0; Yes = 1 | 0-1 | Vaccination uptake, defined as receiving at least one dose |
| Source of HPV vaccine knowledge | A11 | Five nominal categories | Categorical | Descriptive variable |
| Active information seeking | A12 | No = 0; Yes = 1 | 0-1 | Active searching for HPV vaccine information |
| HPV vaccine knowledge | K1-K9 | Correct = 1; incorrect/uncertain = 0; summed score | 0-9 | Higher scores indicate greater HPV vaccine knowledge |
| Perceived susceptibility | HB1-HB3 | Mean of 3 Likert-type items | 1-5 | Higher scores indicate greater perceived susceptibility |
| Perceived severity | HB4-HB6 | Mean of 3 Likert-type items | 1-5 | Higher scores indicate greater perceived severity |
| Perceived barriers | HB7-HB9 | Mean of 3 Likert-type items | 1-5 | Higher scores indicate greater perceived barriers |
| Perceived benefits | HB10-HB12 | Mean of 3 Likert-type items | 1-5 | Higher scores indicate greater perceived benefits |
| Self-efficacy | HB13-HB14 | Mean of 2 Likert-type items | 1-5 | Higher scores indicate greater perceived self-efficacy related to HPV vaccination |
| Vaccination intention | VI1-VI4 | Mean of 4 Likert-type items | 1-5 | Higher scores indicate stronger HPV vaccination intention |
| Acceptable vaccine price | B1 | Five ordered categories | 1-5 | Higher values indicate higher acceptable price |
| Reasons for non-vaccination | B2 | Multiple choice | Multiple responses | Used descriptively if analyzed |
| Social support and perceived service accessibility | SS1-SS4 | Mean of 4 Likert-type items | 1-5 | Higher scores indicate greater social support and perceived service accessibility |
| E-health literacy | EHL1-EHL8 | Mean of 8 Likert-type items | 1-5 | Higher scores indicate greater e-health literacy |

**Reliability statistics reported in the manuscript**

| **Scale/Dimension** | **Items** | **Reliability Statistic** | **Value** |
| --- | --- | --- | --- |
| HPV vaccine knowledge | 9 | KR-20 | 0.780 |
| Perceived susceptibility | 3 | Cronbach's alpha | 0.836 |
| Perceived severity | 3 | Cronbach's alpha | 0.915 |
| Perceived barriers | 3 | Cronbach's alpha | 0.886 |
| Perceived benefits | 3 | Cronbach's alpha | 0.880 |
| Self-efficacy | 2 | Cronbach's alpha | 0.799 |
| Social support and perceived service accessibility | 4 | Cronbach's alpha | 0.862 |
| E-health literacy | 8 | Cronbach's alpha | 0.969 |
| Vaccination intention | 4 | Cronbach's alpha | 0.916 |

Note. KR-20 = Kuder-Richardson Formula 20. Confirmatory factor analysis supported the single-factor structure of the social support and perceived service accessibility measure (CFI = 0.97, RMSEA = 0.05).

# Supplementary Table 3. Between-Profile Comparisons and Effect Sizes

**Continuous variables**

| **Variable** | **High-Barrier/Moderate-Benefit Profile** | **High-Benefit/Low-Barrier Profile** | **Test Statistic** | ***p* Value** | **Effect Size** | **Interpretation** |
| --- | --- | --- | --- | --- | --- | --- |
| HPV vaccine knowledge | 4.61 (2.96) | 6.54 (2.19) | t = -18.378 | <0.001 | Cohen's d = 0.74 | Medium-to-large difference |
| Social support and perceived service accessibility | 3.26 (0.76) | 3.55 (0.78) | t = -8.675 | <0.001 | Cohen's d = 0.33 | Small-to-medium difference |
| E-health literacy | 3.51 (0.75) | 4.00 (0.68) | t = -16.385 | <0.001 | Cohen's d = 0.69 | Medium-to-large difference |
| Vaccination intention | 3.43 (0.73) | 3.96 (0.71) | t = -17.048 | <0.001 | Cohen's d = 0.73 | Medium-to-large difference |

**Categorical variables**

| **Variable** | **High-Barrier/Moderate-Benefit Profile** | **High-Benefit/Low-Barrier Profile** | **Test Statistic** | ***p* Value** | **Effect Size** | **Interpretation** |
| --- | --- | --- | --- | --- | --- | --- |
| Heard of HPV vaccine | 1757 (86.1%) | 684 (95.7%) | chi-square = 46.744 | <0.001 | Cramer's V = 0.13 | Small effect |
| Actively searched HPV vaccine information | 1036 (50.8%) | 502 (70.2%) | chi-square = 80.227 | <0.001 | Cramer's V = 0.17 | Small effect |
| Received HPV vaccine | 312 (15.3%) | 176 (24.6%) | chi-square = 30.924 | <0.001 | Cramer's V = 0.11 | Small effect |

Note. Values for continuous variables are presented as mean (standard deviation). Values for categorical variables are presented as n (%). Effect size estimates were added to help distinguish statistical significance from practical magnitude in the context of a large sample.

# Supplementary Figure 1. HBM-IMB Integrated Theoretical Framework

Note. HBM = Health Belief Model; IMB = Information-Motivation-Behavioral Skills model. The HBM dimensions were used as indicators for latent profile analysis, whereas IMB-informed variables were used as correlates of profile membership.

# Supplementary Figure 2. Study Recruitment and Analysis Flowchart

Note. The valid questionnaire rate was calculated as the number of valid questionnaires divided by the number of collected questionnaires.
